# Collectanea

**Published:** 1827-08

**Authors:** 


### COLLECTANEA.

Floriferis ut apes in saltibus omnia libant,
Omnia nos, itidem, depascimur aurea dicta.

### ANATOMY.

*Account of a remarkable Production, resembling a Tail, which was attached to the extremity of the Vertebral Column of a Man*; by ARTHUR JACOB, M.D.—I AM indebted to my father for the specimen of remarkable monstrosity which forms the subject of the present notice. In the course of last summer, a young man presented himself at the Queen's County Infirmary, seeking relief on account of a tumor which projected between the nates, and caused much inconvenience from its bulk, especially in the sitting posture. It had existed from birth, was about the size of the closed hand of a large man, and was situated upon the lower part of the sacrum, apparently involving the os coccygis. Upon its most convex part was an orifice, through which the finger could be introduced, and passed round an irregular resisting body, which partly projected through this opening. The surface was covered by sound skin, furnished with a considerable quantity of hair. The operation of removing it was performed by my father. On dividing the external layer, it appeared that this was merely a pouch of skin reflected inwardly, and embracing a cylindrical resisting body, nearly six inches long, and thicker than the thumb. This cylindrical body was doubled upon itself, as if so disposed in order to fit in the pouch; it also was covered with true skin. This extraordinary production adhered firmly by one extremity to the spine, and, upon attempting to detach it, the connexion was found to be osseous. The bone, however, was so spongy that it yielded to the knife. The superfluous skin having been removed, the lips of the wound were brought together, and it healed satisfactorily, leaving the patient free from distress on this account.

When the pouch was first divided, and the contained member fully displayed, its appearance was so characteristic that no doubt was entertained of its being a real tail; which has since caused no small degree of merriment. When the part came into my possession, I made a longitudinal section of it from end to end, and found that it was furnished throughout with a bony core or centre, consisting of separate pieces, distinctly joined to each other with perfect synovial capsules.

That it resembled a tail in its external characters I admit; but dissection proves that the bones which occupy the centre bear no resemblance to vertebræ, but, on the contrary, that they are such as might belong to a man's great toe; consisting of a tarsal bone, (probably the internal cuneiform,) a metatarsal, and phalanges. It, in fact, appears to be an example of that description of monstrosity which has often been observed in other situations,—a rudiment or portion of an additional limb or extremity. This one is, however, remarkable from its consisting of but one series of bones out of the five, as well as from the situation which it occupied. (*Dublin Hospital Reports.*)

### PHYSIOLOGY.

*Remarks on the Human Voice*; by D. Liskovius, of Leipsig.—The remarks by M. Liskovius are in the form of a reply to objections advanced by M. Rudolphi against previous statements and opinions of the former. M. Liskovius had said, that the vocal cords lengthened as the aperture of the glottis dilated. M. Rudolphi remarked upon this, that during ordinary inspiration and expiration, the glottis is so much open that no sound can be heard; and, consequently, that, to produce even the lowest notes of the gamut, a dilatation greater than this cannot be required, as it would have no other effect than to prevent the formation of sound altogether. In reply, M. Liskovius remarks that, when in a state of repose, the larynx holds a middle place between its greatest elevation and depression, and that the glottis also is an intermediate state between its greatest contraction and extension; the more the larynx descends, the more the glottis is dilated, and the greater the degree in which the former ascends, so much the more is the latter contracted: if, therefore, the glottis were the most dilated in a state of repose, the larynx should, at the same time, be at the maximum of depression, and its depressing and dilating muscles would be at the maximum of contraction: in such a case, each person would talk in the lowest tones of his voice; as the state necessary to produce them would be that which would offer itself.

That ordinary respiration produces no sound, does not depend upon the too dilated state of the glottis, but upon the feeble impulse given to the column of air traversing the larynx; a point neglected by M. Rudolphi. As to the falsette, it is produced by the anterior part of the glottis, the ligaments being at such time tense and drawn together, whilst the open or pectoral voice is produced by the approximation of the open cords, without any simultaneous tension. M. Liskovius contends also that the falsette is a well-characterised species of voice, and not an imperfect voice, as has been supposed by Haller, Kempelin, and Rudolphi. The conclusions drawn, and principles established, are founded upon anatomical examinations of, and experiments upon, the human body. (*Journal of Science*, from the *Archiv. für Anat.*)

*On the Exhibition of Medicines by the Skin.*—M. Bally, physician of La Pitié, continues to experiment on this subject. He considers that, as obstacles frequently present themselves to the internal exhibition of medicines, we ought to seek some other plan. The epidermis would, in most instances, prove a great obstacle to the introduction of medicines through the skin: this therefore is removed by the previous application of a blister, to permit more free absorption. The following are some of the principal results.

The Salts of Morphine quickly show their influence on the brain and nervous system. The pupils contract, the eyes become brilliant, dysuria and ischuria are sometimes induced; nausea and vomiting rarely. A sense of itching exists to a troublesome degree, chiefly in the nasal fossæ, and frequently numerous papulæ appear on the skin.

The Extract of Belladonna, rubbed on the dorsal side of the feet, produces all the effects which result from it when internally prescribed; such as extreme dilatation of the pupils, and a diminution of the powers of vision.

The Extract of Squills, at the same time that it produces diaphoresis, favours the urinary secretion and promotes expectoration.

Strychnine, well powdered, encourages the suppuration of wounds, and affects the locomotive system, but does not excite great nervous perturbation. M. Bally remarks on the employment of strychnine, that sometimes it causes a sensible turgescence in the vessels of the head, a deep colouring of the face, which demands either its suspension or bleedings.

Several of the preparations of Mercury are usefully employed through the medium of the skin. The *endermic* method of administering remedies becomes necessary in cases where the entrances of the alimentary canal are shut up by spasm, as in hydrophobia, tetanus, and some cases of hysteria; or when the stomach revolts against the necessary medicines.

---

### PATHOLOGY.

*A Case of Ruptured Cœcum, which terminated in Death forty-eight Hours after the Accident;* by JOHN SPEER, Surgeon to the Essex Convict Hulk, Kingstown, Dublin.—April the 8th, 1824. I was called on at seven o'clock this morning to visit James Burn, aged thirty-five, strong and muscular. I found him labouring under the following symptoms; violent pain in the abdomen on pressure, chiefly below the umbilicus: constant vomiting of a yellowish fluid; cold extremities; without pulse at the wrist; retention of urine; countenance contracted. His bowels had not been opened for forty-eight hours.

The account I got of this man's complaint, from himself and his wife, was as follows:—He left home the day before with a funeral, which he accompanied for five miles; and, on his return in the evening, he commenced wrestling with a neighbour, and after a severe struggle succeeded in throwing him to the ground; his antagonist fell on his back, with his knees bent upwards, and Burn fell nearly at the same moment with his abdomen on his antagonist's knees, receiving a violent contusion chiefly in the region of the umbilicus. According to his own account, he felt as if something had given way internally; he fainted immediately, but soon recovered, complaining of severe pain in his abdomen, with vomiting. In this state he was conveyed home in an open cart, a distance of five miles, during which the vomiting and pain continued; but, in consequence of his having been intoxicated, no medical aid was obtained until the next morning, although the pain and vomiting were very distressing during the night.

On my first visiting him, every symptom indicated his approaching dissolution; and, although I was of opinion that mortification had taken place from injury of some of the abdominal viscera, yet, being aware of the deceitful state of the pulse which attends all abdominal inflammations, I attempted in the first instance to take away some blood from his arm; but, before three ounces could be extracted, he fainted, with cold sweats about his head and neck. He afterwards was placed in a bath, of the temperature of ninety-six, which gave him some relief; he appeared refreshed, and could turn in his bed with greater ease; the superior part of his abdomen, along the margin of the ribs and across the region of his stomach, could bear pressure without pain; he did not complain of his chest; his respiration was natural, and intellects clear; he talked rationally, and gave a very accurate description of the accident; but the distress and vomiting continued; the pain and tenderness about the umbilicus was unabated, and the pulse imperceptible, even at the elbow. He now complained of a great desire to make water, with inability to void it, and pain in the region of the bladder: the catheter was introduced, and a pint of urine drawn off; a large blister was applied to his abdomen, injections were administered, and calomel given, but immediately rejected.

The above symptoms continued until the evening of the 8th, after which he complained of no pain, except when his abdomen was pressed on; his extremities at the same time grew cold, and his countenance became gradually more contracted; his senses were clear to the last, and he was free from hiccup; he expressed himself relieved, and entertained to the last hopes of a recovery. He died on the evening of the 9th, at six o'clock, forty-eight hours after he met with the accident.

Leave having been obtained to examine the body, the dissection was made in the presence of Mr. Adams, when the following appearances presented themselves :—The abdomen being opened, a quantity of the contents of the intestines was found in the cavity, and, when pressure was made over the large intestines with the hand, their contents were forced through an aperture in the intestines; aud, on further examination, the cœcum was found ruptured. The aperture was about two inches in circumference, with uneven ragged edges, and evidently the consequence of the fall he had received; it was surrounded with marks of extensive inflammation, as were all the small intestines, on whose surface several layers of coagulable lymph had been deposited in different places, forming a false membrane of a soft texture.

The correspondence of the symptoms which this case presented with those which, in the latter stages of enteritis, follow the perforations of the intestines, and the escape of their contents into the cavity of the abdomen, is well worthy of observation. *(Dublin Hospital Reports.)*

*Case of remarkable Pulsation in the Veins.* By CHARLES DAVIS, M.D. Member of the Royal College of Surgeons, and Surgeon to the North-East Dispensary, Dublin.—On the 21st June, 1826, I was desired to visit Margaret Connor, aged six years, residing at No. 49, Clarendon-street, whom I found labouring under symptoms of acute hydrocephalus.

She was much emaciated, and, upon inquiry, I learned that four months previously she became affected with hooping-cough, from which she had never perfectly recovered; and that, within the last ten days, she had been suddenly attacked with a vomiting of bilious fluid, which still continued.

She now appeared listless, and regardless of surrounding objects; her tongue was coated with a yellowish fur; she had complete loss of appetite for solid food, but took drink when presented to her; and, when questioned, complained of dull pain in the forehead; her pupils were somewhat dilated, but readily contracted upon the admission of light; her skin was hot and dry, and her pulse, which was somewhat irregular, beat eighty-eight in the minute, and was full and pretty strong; her bowels were costive, and her stools, when procured, green and hydrocephalic.

An eruption of dark spots, larger than petechiæ, somewhat resembling purpura simplex, was scattered over the skin; and, upon the chest and neck, a miliary vesicular eruption was observable.

What surprised me much in this case, and now induces me to lay it before the profession, was a pulsation in all the veins, distinct and well marked, synchronous with that of the arteries, and in the veins of the extremities, perceptible to the eye, even at the distance of two yards.

The veins were rather larger than is usual at her period of life; and pressure upon any of them stopped the pulsation between the part compressed and the heart, so that it obviously could not be caused by regurgitation from the auricle.

The pulsation in the part of the vein towards the extremity was rendered much stronger and more distinct, provided the return of the blood to the auricle was not completely obstructed; but if the compression was so strong as entirely to obliterate the calibre of the vein, that part of it, which became tense and distended with blood so far as the next valve, after a few seconds lost the pulsation altogether.

The pulsation of the heart was somewhat stronger than usual at her age; the pulsation of the veins was softer than that of the arteries, and was completely stopped on compression of these latter vessels.

In despite of leeching, cold applications to the head, and blisters, combined with the exhibition of calomel, constantly repeated in minute doses to enable it to remain upon the stomach, and every other remedy which suggested itself to my mind, or was recommended by those of my medical friends who saw the case with me, (amongst whom was Mr. Peile, who kindly lent me his assistance,) it terminated fatally upon the 28th of June; on the evening of which day I made an inspection of the body, of which the following is an account:

The vessels of the pia mater, particularly towards the base of the brain, were somewhat fuller of blood than is natural; and the substance of the brain little, if at all, softer than is usual at this period of life.

The ventricles contained at least four ounces of transparent fluid, and the choroid plexus seemed pale, as if macerated.

The pericardium contained a small quantity of serous fluid, and the left ventricle of the heart was somewhat enlarged, and firmer than natural; all the other viscera of the thorax and abdomen were healthy.

The arteries had been injected, but presented no preternatural communication whatever with the veins; neither could any artery be discovered in the immediate vicinity of these latter vessels, which could throw a doubt on the fact that the pulsation had been continued from the heart, through the arteries and capillaries, to the veins. *(Ibid.)*

*Consequences of Wounds received in Dissection.* By A. Colles, M.D.—On Tuesday, May 18th, 1824, Mr. Shekelton was engaged in examining the body of a man who had died of peritoneal inflammation, consequent on the operation of lithotomy. The examination took place in a very few hours after death, the body still retaining its heat. I observed that, soon after the abdomen was opened, Mr. Shekelton pricked himself with the point of the knife, which called forth the usual involuntary expression of pain, but was not further attended to. He proceeded to take out the contents of the pelvis, so that his hands were necessarily immersed in that cavity for a considerable time.

On the following evening (19th) he felt himself unwell; on Thursday (20th) he gave an anatomical demonstration as usual, and immediately after it he went home very ill, but, owing to the natural reserve of his disposition, he did not apply to any of his medical friends. On Friday evening (21st) Mr. Cusack accidentally saw him, and found some of the glands swelled in the left axilla, from which he was apprehensive that the disease was produced by the wound received on Tuesday. When Mr. Cusack again visited him on (22d) Saturday, the condition of these glands was so much improved as to induce him to look upon the case as one of simple fever brought on by cold and fatigue, to which Mr. S. had lately been much exposed.

On Saturday morning (23d) I first saw Mr. S.: he told me, in a most emphatic manner, that he had passed a most wretched night; yet the febrile symptoms were not very severe. Pulse only eighty-four, though his skin was hot, and there was an indescribable anxiety and distress very perceptible. He said that he felt as if his stomach was too full. The glands in the left axilla were slightly enlarged, and tender to the touch; but there was neither swelling nor redness along the arm. He complained of pain along the course of the ulnar nerve, and down the left side of the thorax. There was no appearance of inflammation in the ring-finger, which had been wounded: indeed, we could scarcely discover any trace of the wound. Were it not that I recollected to have seen him wound the finger, and that his present anxiety and distress very far exceeded in severity the other febrile symptoms, I should have supposed him affected with common fever only.

By his own desire, leeches were applied to the axillary glands; but he suffered so much from the bites of each of these, that, after the fourth had fixed, he refused to let any more be applied. He took an emetic, which operated well, but did not produce any marked impression on the symptoms.

Monday (24th.)—He complained of uneasiness in the stomach and bowels, and felt as if he would be much relieved by having a free stool. His tongue was covered, but not very thickly, with a white mucous coating; skin of the face assuming a yellow colour; features rather sharp; no uneasiness in the course of the lymphatics of the arm; swelling and pain of the axillary glands had ceased: so that some of his medical friends still indulged the hope that the case would prove to be but a severe instance of common fever.

Tuesday (25th.)—Constitutional distress increased, and the real nature of the disease was too obvious to all his medical attendants. We agreed that he should have a full opiate at night.

Tuesday evening.—This afternoon a red spot, the size of a shilling, appeared on the right patella. For two hours this evening he suffered such severe torture in the knee, that he declared he would much rather endure the pain of having every limb amputated in succession, than again undergo the pain he had suffered in that knee. This pain he referred to the cavity of the joint.

Wednesday (26th.)—At ten o'clock last night, he got sixty drops of laudanum, and in the course of two hours twenty more: these produced very profuse and general perspiration; but no alleviation of symptoms followed. He dozed a good deal, and his attendants reported that he had passed a good night: he himself declared that it was a most uncomfortable night.

During the night his brother, Dr. Robert Shekelton, observed a large, red, and swollen patch over the right tibialis anticus muscle. This, and the spot on the patella, had now a solid feel, and were not very tender to the touch.

Early this morning (at two o'clock) the right arm, from the shoulder down to the elbow, was observed to be swelled, but without discoloration; the right thigh also was swelled, but not discoloured. A red patch appeared on the dorsum of the left foot, and he complained of a pain in the left scapula and shoulder. Tongue covered with a thick coating of white mucus; countenance of a deeply yellow tinge.

Thurday (27th.)—He now complained of pain of his left arm, with considerable swelling of it and of the forearm. Yellowness of skin was now universal, and more deep than before; adnatæ of the eyes very yellow; countenance more sharp and contracted; tongue as yesterday.

At six o'clock this evening, his weakness became extreme, his features very sharp and contracted; yet his pulse was regular, and not very quick.

Friday (28th.)—Twice in the course of last night he was supposed to be dying; yet he soon after called for solid nourishment, ate a teacupful of panada, and took the yolk of an egg beat up with brandy: this he did from some notion that he now only required solid food in his stomach to complete his recovery. He was obviously not free from delirium.

During the night he passed a large quantity of urine, of a peculiarly dark brown colour.

At five o'clock this morning he sent for me, and requested that I would make incisions into the left arm and forearm, which were much swollen, but free from redness: indeed, the only place where any redness could be seen was on the left scapula. I could not refuse to comply with his wishes, although I knew how unavailing this measure must be; for the hand was livid, and cold as a gangrened hand; he had the power of moving the thumb only; all his extremities were cold; this was the coldest; the right arm and forearm were less swelled and soft; the spot on the middle of the right leg, which always had a solid feel, was now more extended and less raised.

I made an incision into the red patch on the back of the left shoulder, on the inner side of the arm, on the forearm, and along the outer edge of the biceps at the bend of the arm. All these incisions were carried through the fascia of the limb. Nothing, except a small quantity of blood, issued from any of these incisions: in none of them did the skin or cellular substance exhibit any diseased appearance, except in that at the bend of the arm, where the cellular membrane had a deep yellow colour. His brother, in the course of the night, had punctured the bursa on the olecranon of the left arm, which was very tense, red, and painful: from this a large quantity of red watery fluid still continued to flow. During the night he complained that half a glass of claret felt as strong and hot in his stomach as if it had been ardent spirits.

He died at nine o'clock this morning.

In this case we recognise the same train of symptoms as in the cases of Mr. Hutchinson and Mr. Dease, reported in vol. iii. of Dublin Hospital Reports, &c. The present case tends to strengthen the opinion I then advanced—"that slight wounds, received in dissecting fresh bodies, sometimes give rise to a peculiar disease, perfectly distinct from every other disease consequent on similar wounds." In addition to the other symptoms there described as being characteristic of this disease, we may mention this striking one,—viz. that previously to the disease terminating either in death or in recovery, swelling and inflammation seize upon the portion of the limb interposed between the original wound and the first seat of pain. We see that this took place in Mr. Hutchinson's case at so late a period as three weeks; the fever, although it had remitted, not having ceased until this inflammation had occurred. In the fatal cases of Mr. Dease and Mr. Shekelton, it appeared, in the former on the ninth, in the latter on the tenth day.

I must entreat the attention of the reader to this fact, that the redness which is seen on the swollen parts is very unlike that of erysipelas; for the colour is that of a peach-blossom, is of very small extent compared with the extent of the swelling, is seen for a few days, perhaps for a few hours only, on the same spot, and next is observed in some very distant part, possibly in the opposite limb: besides, this peculiar redness vanishing quickly from a part does not leave any vesication or desquamation after it, as is seen in cases of erysipelas.

If any proof be wanting to establish an essential difference between this disease and phlegmonoid erysipelas, it will be found in the state of the swollen parts when cut into. For, although four incisions were made into different parts of the left arm of Mr. Shekelton, yet no discharge, except a small quantity of blood, issued; nor was any change of structure visible except that slight one which has been noticed at the bend of the arm. *(Ibid.)*

THE following *Case of Aneurism of the Aorta* is related by Dr. COMSTOCK, in a recent Number of the *Philadelphia Journal.*

Hester Hopkins, a negro woman, aged seventy-two years, died suddenly on the 17th of December, 1826, in consequence of a profuse hemorrhage from the bowels. I did not see her till after her death; but I received from her daughter and attendants the following history of her case:—

About seven years ago, the deceased was engaged in spreading peaches upon a platform, when suddenly her foot slipped, and she fell with her abdomen directly across a fence. From that period to the time of her death, she was subject to pain in her abdomen, whenever she took cold, or was a little unwell. It did not, however, interfere much with her work, as she has spent most of her time at hard labour since the occurrence of the accident.

About nine months previous to her death, she first observed a pulsating tumor in her left side, and from that period was subject to constipation.

On the evening of the 15th, feeling somewhat unwell, she took a dose of rhubarb, which operated several times, bringing away feculent discharges, but no blood. The operation of the medicine afforded her some relief.

On the 16th, the tumor pulsated violently, and was very painful. The nurse, supposing it to be an abscess, applied a succession of fomentations as hot as could be borne. About three hours after their first application, the patient said she felt the tumor burst internally. She immediately rose from her bed, and discharged six pints of dark blood, mostly coagulated. This debilitated her exceedingly, but her system soon reacted.

On the morning of the 17th, she felt pretty comfortable: she sat up most of the time, was very cheerful, and laughed and talked with the family. Between four and five o'clock P.M., while lying on the bed, she felt blood flowing into her bowels; she threw back the bed-clothes, got up without assistance, and discharged a pint of florid blood; about the same quantity had previously escaped upon the bed. She now fell prostrate on the floor. She was replaced upon the bed by her attendants, and after moaning a few minutes she expired.

On the 19th, assisted by my friend George Spackman, student of medicine, I examined the body. On opening the abdomen, a large tumor presented itself immediately behind the sigmoid flexure of the colon. It originated in front of the lumbar vertebræ, and extended into the left iliac region. It was firmly attached to the muscles behind it, and was connected with the left ovarium, apparently by adhesive inflammation. That part of its anterior surface over which the colon passed was identified with the structure of that intestine.

The tumor was of a dark bluish colour, and, when pressed by the fingers, felt like a mass of flesh. On laying it open, it proved to be an aneurism of the aorta. It originated from that portion of the aorta which lies between its bifurcation and the part where the emulgent arteries are given off. It was divided by an imperfect septum. The superior right division was formed by a dilatation of all the coats of the artery. In one-half of this division, the internal coat was wanting, and the surface presented a caruncalated appearance. But there was sufficient evidence that the internal coat had undergone extension, as the width of the remaining portion was much greater than the circumference of the aorta above the aneurism. This division of the sac was nearly filled by a firm lamellated coagulum, which, when cut, resembled tendinous matter: hence it appears that a spontaneous cure had been attempted. The structnre of this coagulum is very well represented by one of the plates in HODGSON's work on Diseases of the Arteries.

The lower division of the sac was formed of cellular substance, and was filled with coagulated blood. But the most interesting part of the investigation was the discovery of an opening, three-fourths of an inch in diameter, at its inferior and anterior part, communicating with the sigmoid flexure of the colon. This at once revealed the cause of the hemorrhage and sudden death of the patient.

After a careful examination of the aneurismal tumor, and a little reflection upon the circumstances of the case, I am inclined to the opinion that the superior division of the sac, which might be termed the *primary* aneurism, was of long standing, and probably originated from violence done to the aorta, when the patient fell upon the fence. At a subsequent period, perhaps about the time the patient first observed a tumor in her side, ulceration occurred in the left portion of the sac, or some violence was done to it, which caused it to rupture, and an opening was formed, allowing blood to be effused between the muscles and the peritoneum; and thus a *secondary* aneurism was produced. This mass of blood, lying behind the sigmoid flexure of the colon, acted as a morbid irritant to that intestine; ulceration was the consequence, and a communication was formed with the sac, through which the hemorrhage took place, that terminated the life of the patient.

Cases of this description, I believe, are rarely met with. One instance, somewhat similar, is noticed in "The Lectures of Sir ASTLEY COOPER." The aneurism, however, was higher up, and communicated with the jejunum. It is a curious circumstance, that in that case also the patient survived till the day after the first discharge of blood.

---

### SURGERY.

M. LISFRANC'S *Signs by which the Presence of Pus may be in general detected.*—He advises to apply on the part the three middle fingers of each hand, separated about an inch from each other; then with the left hand make pretty strong pressure; the right hand, remaining fixed, will feel the liquid which distends the portion of the cyst placed under it. In its turn the right will make pressure, whilst the left hand will remain fixed, and so on.

This method, which is very good when the abscess is large and superficial, might lead into errors when the matter is small in quantity and deep-seated. In the same way, when the disease is situated in the soft tissues, offering little resistance,—such, for example, as those of the mammæ, of the abdominal parietes, of the thigh, &c.—the percussion communicated to these parts by pressure might stimulate fluctuation, and give birth to mistakes. In such cases, let the fluctuation be sought for after the following manner:

Apply the three middle fingers of the hand on the point where we suspect a purulent collection; make a smart and pretty firm pressure on it; then suddenly desist, but without removing the fingers. The pressure made may be arrested by a hard body, which is the anterior wall of the cyst applied against the posterior. The matter displaced by the pressure regains its place the moment pressure ceases, and the fluctuation is easily recognised. If the cyst is not very full, a kind of gurgling or undulation may be felt, which it is sufficient to have once perceived to prevent the risk of future mistake.

There is a third method by which fluctuation may be felt. It consists in placing one hand on one side of the tumor, and making gentle percussion with the other on the opposite side. After each percussion, a rush of matter is felt to strike the hand resting on the tumor.

These are not the only means that practitioners should make use of to recognise the presence of matter: there are some very important modifications to be put in practice, according to the seat of the abscess.

When the purulent collection is in the midst of very mobile textures, which do not offer any point of support, or if it be very deeply situated,—such as the mamma, the walls of the abdomen, the thigh, the calf of the leg, &c.—let the hands of an assistant be closely applied to the part, that it may be rendered perfectly steady; otherwise, the sort of vibration arising from the mobility of parts is apt to deceive.

2. When purulent collections take place in the orbit, cases occur in which it is difficult to recognise a fluctuation. The tissue of the eyelids, at that time always infiltrated with serosity, and the great mobility of the eye itself, lead into error: added to these two circumstances, the cellular texture in this cavity being very loose and permeable, the matter often breaks down a much greater space than is necessary to contain it. Let us attend to the following direction:—The patient closes both eyelids; we make pressure on them in front: by this means the eye is pushed back into the bottom of the orbit, the pus is concentrated towards the base of the cavity, projects under the inferior eyelid, and the fluctuation is easily felt.

3. When an abscess developes itself outwardly at the lower and external part of the auditory canal, if the collection is voluminous, no mistake can possibly be committed; but if the collection is not great, the pressure made to ascertain its presence causes it to impinge against the external auditory canal, which is raised up: it is then very easily mistaken. In this case, introduce a female sound into the passage of the ear, depress gently with this instrument the lower part of the cavity. By this means the matter is pushed out externally, the textures are fixed, and the fluctuation is then easily felt.

4. Does the matter exist in the moveable parietes of the mouth? the practitioner, in order to steady the textures, should put one or two fingers into that cavity to push them outwards, while with the other hand he feels the fluctuation.

5. *Abscess situated under the scapula.*—A woman, brought to bed recently in La Pitié, had a purulent collection under the shoulder blade, which was recognised by the following signs:—The scapula was raised; when pressure was made upon it, and it was depressed, making the fluid shift its place, in the same manner as, in a dropsy of the knee-joint, the patella is depressed when the limb is extended. The pressure made upon the shoulder-blade forced a part of the matter to the margin of the bone, more particularly towards the inferior angle, and the external and spinal borders. Towards these points small bands overlap, and disappear when pressure ceases.

Instead of trepanning the shoulder-blade, as Marechal did in a similar case, M. Lisfranc gave exit to the matter by an incision made immediately under the inferior angle of the bone; and, in cases where the matter is situated more deeply under it, the same incision might be made, and then a catheter introduced into the cyst, which would easily reach it.

6. When an abscess appears in the neighbourhood of the vulva or anus, we should, to avoid mistake, introduce one or two fingers into these cavities as far as possible; press from above downwards, and from within outwards, upon the painful point. By this means the tissues are made steady, and the matter projects outwards.

7. When matter is secreted in the perineum, under the perineal aponeurosis, it is then impossible for it to project outwards: it is in examining by the vagina or rectum that we shall be able to discover the matter through the adjacent mucous membrane.

8. Is the abscess situated about the femero-tibial articulation? It is difficult, in some cases, to distinguish it from dropsy of the joint. However, it is of the greatest consequence that they should be distinguished, because in the collection of pus an opening should quickly be made: in dropsy of the joint, it is well known how carefully an opening is to be avoided.

With regard to the opening of abscesses, it will suffice to say that M. L. uses the knife. When he has to open any extensive abscesses, lays them open by little bits: he makes an incision one or two inches long, waits a day or two till any febrile symptoms pass away, and then makes another similar incision, and so on till the whole sac is laid open; and it has frequently happened that, as the incisions were continued upwards, the lower cuts cicatrised. As an example of this, a young man, aged eighteen, of lymphatic temperament, came into La Pitié, April 3d, 1825: he had a fluctuating tumor, larger than two fists, in the lumbar region; it mounted up as far as the inferior angle of the scapula, and was at least three inches broad; he had never felt pain any where but in the situation of the tumor. On the 7th, the tumor was punctured, and a flocculent ill-digested pus given vent to: the pus was evacuated gradually.

20th.—The sac was completely emptied; it preserved nearly the same dimensions. It was in vain that all means of compression were used with it till May 2d; at that time a probe passed up nearly five inches. Neither emollient nor irritating injections produced any amendment.

May 10th.—M. L. laid open to the extent of two inches of the sac. Charpie was introduced between the lips of the wound. Inflammation, with some fever, came on, which soon subsided by rest and diet.

18th.—An inch and a half more was laid open.

24th.—Another incision laid open the upper point of the sac; but, as the sac extended into the flank, after some time incisions similar to those described were practised, and with the most complete success.

*Chronic Enlargements.*—M. Lisfranc tells us, before attempting to cure any chronic enlargement, it would be well to examine carefully the condition of the thoracic and abdominal viscera, &c. When any advanced organic alteration is recognised, the radical cure of the external affection ought not to be attempted. It has often been observed that the visceral disease becomes aggravated as the other diminishes. Cure the visceral affection first, and then the chronic enlargement. As often as pain (no matter how slight) shows itself, apply thirty or forty leeches, being regulated by the strength of the patient as to the quantity of blood that should be taken. Reduce the diet of the patient, and cover the part with emollient poultices. If the sub-inflammation resists the first local bleeding, it must be reiterated as the constitution of the patient will bear it.

To produce a complete cure of the disease, recourse must be had to excitants and resolvents. Among the first, M. L. ranks the applications of small numbers of leeches, (from two to six, and with the precaution not to encourage the flow of blood.) Their action in this case is exciting: in hundreds of cases at La Pitié, M. L. has proved this fact. After such an application of leeches, we see the next day the volume of the tumor is increased, and its sensibility also; sometimes there is an erysipelas. But these symptoms of increase need not excite alarm. In forty-eight hours after, the erysipelas, if slight, is gone; the swelling and sensibility diminish; and the volume of the tumor is reduced,—if not, its consistence is softened. Four or five days after, a new application of leeches is indicated, and so on till the cure is completed. Should the degree of inflammation produced run to a great height, then it must be subdued by a numerous application of leeches. Scarifications, M. Lisfranc thinks, are sometimes useful. His mode of scarifying is to make twenty or thirty punctures with the point of a lancet or straight bistoury, and to leave between each nearly a third of an inch, so that the inflammatory circle which forms round the one shall not join the others. The tumor is to be covered with an emollient poultice.

*Case of Abscess of the Liver, in which an Incision was made nearly down to the Matter.*—A robust man, by trade a glass-blower, was admitted into the new Meath Hospital; he laboured under well-marked symptoms of acute inflammation of the liver. Although very active means were used, complete resolution of the inflammation was not induced, and, for four weeks after the subsidence of the first attack, the symptoms left no room to doubt the formation of an abscess in the liver. Hectic fever, attended with rigors, night sweats, and emaciation, being accompanied by a constant sense of uneasiness and weight in the right hypochondrium, which was evidently enlarged and harder than natural. It was also tender and painful at first; but after some time the pain became confined almost to one spot, which nearly corresponded with the centre of the external elevation.

Poultices were diligently applied; but, although a very indistinct feeling of deep-seated softness was soon perceptible to the touch, yet the abscess showed no tendency to point outwards. The external swelling remained stationary, and the integuments were of a natural colour. The man's constitution was now rapidly giving way, and it therefore became a most important question, whether the abscess in the liver should be opened by an operation? To the performance of an operation it was objected, that the external tumor was very diffused, and of course the situation of the abscess quite uncertain, so that an operation afforded but little chance of giving exit to the matter; and if it failed, it might, for obvious reasons, prove very detrimental. Any attempt, therefore, to open the abscess was disapproved of by the surgeons of the hospital.

Under these embarrassing circumstances, it occurred to me that I had seen several cases where an incision made over a deep-seated abscess had failed, from its deep situation, to give vent to the matter in the first instance, and yet, in the course of a few days, the abscess found its way to the incision, and burst through; a process explicable partly by the removal of pressure, and partly by the inflammation arising from the incision, and which served to form a connexion between it and the abscess.

On these grounds I proposed that an incision, about four inches long, should be made exactly over the centre of the tumor in the right hypochondrium: that it should be carried through a considerable depth of muscles, and, if possible, be continued to within about one or two lines of the peritoneum.

This incision was to be plugged at its bottom with lint, and thus kept open, in the hopes that the hepatic abscess might, for the reasons above mentioned, tend towards and finally burst through it. The operation was performed by my colleague, Mr. Macnamara. The abdominal muscles were found of considerable thickness, and quite healthy; and, although the incision was very deep, yet the situation of the hepatic abscess was not felt more distinctly, so that it now became quite evident that no prudent surgeon would have persevered in an attempt to open directly into it.

I now waited for the result with much anxiety. In two days after, the patient sneezed, and purulent matter in very large quantity burst forth through the wound. On examination, it appeared that the incision had not been made exactly over the abscess in the liver, for the matter did not come from the bottom, but from one side of the wound, and pressure on the liver to that side caused matter to flow in abundantly. The communication between the wound and the abscess was not therefore directly inwards, but somewhat laterally. If then we had attempted to open the abscess *directly*, we would have failed, and the consequences of such an attempt might have been fatal. Purulent matter, at first in large and afterwards in diminished quantity, flowed through the wound for several weeks, and the man perfectly recovered.

The mode which was so successfully adopted in this case is, I believe, novel, and may prove serviceable in similar cases, many of which have hitherto been considered beyond the reach of art. Its safety is at least a strong recommendation. I have little doubt that its judicious employment may be the means of saving many lives, particularly in the East Indies, where suppuration is so frequent a consequence of hepatitis.

(The above case is related by Dr. Graves, in the *Dublin Hospital Reports.*)

*Cases of the Excision of Carious Joints*; by Philip Crampton, F.R.S. &c.

The success which in many cases of compound dislocation of the shoulder, elbow, and ankle joints, attended the removal of the protruding extremity of the dislocated bone, led Mr Park, of Liverpool, to entertain the original idea that, in certain diseases of the joints, the excision of the carious extremities of the bones might be attended with similar advantage.

Accordingly, in a case of scrofulous disease of the knee, (commonly called white swelling,) Mr. Park extirpated the whole of the joint, "removing somewhat, though not much, more than two inches of the femur, and of the tibia rather more than an inch." The operation (which was performed on the

2d of July, 1781, in the Liverpool Infirmary,) was attended with the most complete success. "The man lived to make several voyages to sea; he was able to go aloft with considerable agility, and to perform all the duties of a seaman; he was twice shipwrecked, and suffered great hardships, without feeling any further complaint in the limb, and was at last unfortunately drowned by the oversetting of a float in the river Mersey."

A few years after the appearance of Mr. Park's pamphlet, Mr. P. F. Moreau published his valuable "Cases of Excision of Carious Joints." It appears that in the year 1782, (about a year after Mr. Park's publication,) Mr. Moreau, the father, in a memoir presented to the Academy of Surgery of Paris, proposed the excision of the carious joint as an operation which, under certain circumstances, might be advantageously substituted for amputation.

In the year 1786, he communicated to the Academy "an account of an operation in which he removed the head of the humerus and the corresponding glenoid cavity of the scapula, which were carious." In 1789, he addressed to the same Society a memoir explaining his new method of treating carious joints." This essay, says his son, though supported by many facts, met with the most violent opposition; it was found more convenient to deny than to examine the facts on which it was grounded. "This, however, (he adds,) did not discourage my father, nor did it stop him in his career." Accordingly, "on the 17th of September, 1792, he removed the whole of a carious knee-joint from the son of Mr. Clause, apothecary at Chalons-sur-Marne, in the presence of Mr. (now the Baron) Percy, surgeon-general to the army of Kellerman, of Mr. Chamerlat, his colleague, and of several other eminent surgeons, both civil and military." The operation seems to have been attended with success, for "three months and à half afterwards the wound was healed, and the limb had acquired a considerable degree of firmness; but the Prussians, in retiring from the French territory, left behind them an epidemic dysentery, which, as is well known, carried off the greater part of those who were attacked by it. It got into the hospital at Bar, of which I had the charge, and was communicated to my patient: he could not bear up against it, and on the fifteenth day he died, just three months and a half after the operation."

M. Moreau proceeds to relate several other cases in which the operation of removing the shoulder, elbow, and ankle joints was performed, with complete success, by his father, by Baron Percy, and by himself, and concludes with the following observation: "It was my wish to show, by the evidence of facts, that the excision of carious joints is, in many cases, a very practicable operation, and one that holds out advantages so unequivocal, that amputation ought to be proscribed in every case where excision may be performed."

These were the grounds on which I thought myself justified in reviving the operation of Mr. Park. The following cases will show how far the expectations which I had formed as to the benefits to be derived from the practice have been realised."

Case I.—Alexander Gordon, 90th regiment, aged twenty-three, was admitted into the Royal Infirmary, Phœnix Park, Dublin, on the 2d of January, 1823. To avoid the description of appearances with which every medical man is but too familiar, it may perhaps be sufficient to state that it would be difficult to find an individual in whom the "scrofulous aspect" was more distinctly marked. He was sent to the General Hospital on account of true scrofulous white swelling of the right elbow-joint. The disease was of about ten months' standing; the swelling extended at least a hand's breadth above and below the joint; suppuration had taken place over the inner condyle of the humerus, and the opening had degenerated into a large and irregular ulcer, at the bottom of which the bone could be felt in a state of caries. The man's general health was much impaired: his pulse was 120, and feeble; he had night perspirations, and, in a word, was far advanced in hectic fever. It was determined in consultation that the only chance of preserving his life was by sacrificing the limb, and he was sent into the General Hospital, in order that the operation might be performed. I thought this was a fair case for performing Mr. Park's operation, and having obtained the man's consent, (who declared "that he would willingly suffer any pain or risk for the chance of saving his right arm,") the operation was performed on the 4th of February, in the presence of the greater number of the principal surgeons, both civil and military, of Dublin. The patient was placed (as recommended by M. Moreau) upon his belly on a table, covered with a mattress and pillows so arranged as to make his posture as little inconvenient as possible; the diseased arm hung over the edge of the table, presenting its posterior and inner surface to the operator; the brachial artery being compressed by an assistant, an incision was now made along the spine of the inner condyle, commencing about four inches above, and terminating about two inches below, its tuberosity. This incision passed through the centre of the ulceration, and laid bare the ulnar nerve, which was carefully raised from its groove, and drawn to the inner side of the incision.* A similar incision, parallel to the first, was made on the outer side of the humerus, and then a transverse section, which cut through the tendon of the triceps muscle, immediately above its insertion into the olecranon, connected the two longitudinal incisions, so that the wound represented pretty accurately the letter H; the lateral incisions, however, being slightly incurvated, so as to follow the bend which the forearm made with the arm. The upper flap, consisting of the lower extremity of the triceps muscle, the thickened and diseased cellular substance, and integuments, was raised from the flat surface of the humerus, to which it had a very slight attachment. The lower flap was separated in the same manner, so as to lay bare the upper extremity of the ulna and radius; the scalpel, laid on its flat, was now pushed between the flexor muscles and the bone on its anterior surface, at the distance of three inches above the tuberosity of the inner condyle, and retained in this situation by an assistant. The saw was then applied, and the bone was divided immediately over the flat surface of the knife, which served as a protection to the muscles beneath. The separated portion of the humerus was now raised with the utmost ease by the finger and thumb of the left hand, while the capsular and lateral ligaments, degenerated to the state of a lax cellular substance, were separated by running the knife round the condyles, keeping the edge as closely as possible to the bone. The lower extremity of the humerus being removed, the articulating surfaces of the radius and ulna were completely exposed; but, with the exception of the cartilage which covers the olecranon, (which was partially eroded,) every thing appeared sound. The olecranon was now removed, and the wound was sponged out. As there was no bleeding

* From neglecting this precaution in M. Moreau's case, the ulnar nerve was cut across, and the ring and little fingers were deprived of the powers of motion.

which rendered it necessary to have recourse to a ligature, the flaps were laid down, and secured to each other by four points of suture. The forearm was placed at a right angle with the arm; the wound was covered with pledgets of lint wetted with spirits and water, and the man was laid in bed, with the arm supported on a suitable pillow.

He passed the night remarkably well. Suppuration, attended with a very slight degree of symptomatic fever, set in on the fourth day; but so favourably did every thing proceed, that on the ninth day he sat up in his chair, the arm being supported in a tin case, which I had constructed for the purpose. The wound, however, was slow in healing, no doubt from the bad constitution of the patient: I sent him, therefore, to the sea-side five weeks after the operation, and there he recovered so rapidly that on the following week he walked into town to see me, a distance of nearly five miles. He continued to reside at the sea-side for three months, walking into town and returning on the same day once a-week. On the 18th of September, he returned to the King's Infirmary, in order to pass the board of general officers at the Royal Hospital for his discharge. At this time the wound, with the exception of a small superficial ulceration about the place which had been occupied by the inner condyle, was completely closed; the arm, when allowed to hang by the side, retained nearly a semi-flexed position, but, by a voluntary effort, he was able to give a slight degree of flexion to the forearm, so as to lessen the angle which it formed with the arm. He had the use of the fingers, so as to be able to use his knife and spoon; and, on the 27th of November, 1823, he signed his own discharge with the right hand. *(Ibid.)*

[A second and third case are related, in which the knee joint was excised. In both the patients survived; but in one only was the limb of any use. For further information on this subject, we beg to refer to Mr. Symes's recent cases in the Edinburgh Medical and Surgical Journal.]

